# A Novel lnc-RNA, Named lnc-ORA, Is Identified by RNA-Seq Analysis, and Its Knockdown Inhibits Adipogenesis by Regulating the PI3K/AKT/mTOR Signaling Pathway

**DOI:** 10.3390/cells8050477

**Published:** 2019-05-18

**Authors:** Rui Cai, Guorong Tang, Que Zhang, Wenlong Yong, Wanrong Zhang, Junying Xiao, Changsheng Wei, Chun He, Gongshe Yang, Weijun Pang

**Affiliations:** Laboratory of Animal Fat Deposition and Muscle Development, Key Laboratory of Animal Genetics, Breeding and Reproduction of Shaanxi Province, College of Animal Science and Technology, Northwest A&F University, Yangling 712100, Shaanxi, China; cairui1663@nwsuaf.edu.cn (R.C.); TGR@nwsuaf.edu.cn (G.T.); zhang_que@126.com (Q.Z.); 18821676151@163.com (W.Y.); w19912@sina.com (W.Z.); xiaojunying010306@163.com (J.X.); weichshm@163.com (C.W.); Hc990828@126.com (C.H.); gsyang@nwafu.edu.cn (G.Y.)

**Keywords:** lncRNA, lnc-ORA, adipocytes, proliferation, differentiation

## Abstract

Obesity is closely associated with numerous adipogenic regulatory factors, including coding and non-coding genes. Long noncoding RNAs (lncRNAs) play a major role in adipogenesis. However, differential expression profiles of lncRNAs in inguinal white adipose tissue (iWAT) between wild-type (WT) and *ob/ob* mice, as well as their roles in adipogenesis, are not well understood. Here, a total of 2809 lncRNAs were detected in the iWAT of WT and *ob/ob* mice by RNA-Sequencing (RNA-Seq), including 248 novel lncRNAs. Of them, 46 lncRNAs were expressed differentially in WT and *ob/ob* mice and were enriched in adipogenesis signaling pathways as determined by KEGG enrichment analysis, including the PI3K/AKT/mTOR and cytokine–cytokine receptor interaction signaling pathways. Furthermore, we focused on one novel lncRNA, which we named lnc-ORA (obesity-related lncRNA), which had a seven-fold higher expression in *ob/ob* mice than in WT mice. Knockdown of lnc-ORA inhibited preadipocyte proliferation by decreasing the mRNA and protein expression levels of cell cycle markers. Interestingly, lnc-ORA knockdown inhibited adipocyte differentiation by regulating the PI3K/AKT/mTOR signaling pathway. In summary, these findings contribute to a better understanding of adipogenesis in relation to lncRNAs and provide novel potential therapeutic targets for obesity-related metabolic diseases.

## 1. Introduction

Obesity has become a public health hazard worldwide and is the main cause of cardiovascular diseases, type 2 diabetes, and obesity-associated metabolic syndrome [1]. Adipogenesis is mediated by a series of complex processes, including commitment of mesenchymal stem cells into preadipocytes and the induction of preadipocytes to mature adipocytes [2,3]. The exploration of this process and its regulation mechanism is of great significance for the prevention and therapy of obesity-related diseases. The *ob/ob* mouse is a genetic obesity mouse with a deficiency of the leptin gene that constitutively develops obesity [4]. Therefore, it is a good model for investigating the gene regulatory network of obesity.

Leptin is a hormone that is primarily made and secreted by mature adipocytes and binds to its receptor in the hypothalamus, with positive effects on energy homeostasis and weight loss [5]. Leptin can activate Janus-activated kinase (JAK)-2 and phosphatidylinositol 3-kinase (PI3K) as well as signal transducer and activator of transcription (STAT)-3 pathways to regulate energy metabolism and body weight by increasing proopiomelanocortin (Pomc) expression and inhibiting agouti-related protein (AgRP) expression [6,7]. A previous study identified an obesity-induced long noncoding RNA (lncRNA), lnc-leptin, which regulates adipocytes differentiation through the maintenance of leptin expression [8].

lncRNAs are non-coding RNAs composed of more than 200 nucleotides, which are emerging as regulators of gene expression at the epigenetic, transcriptional, and post-transcriptional levels [9,10,11]. Recently, a growing number of studies have indicated that lncRNAs are critical regulators of adipocytes differentiation, insulin signaling, and browning of white adipose tissue (WAT) [12,13,14,15]. Spi-1 proto-oncogene antisense lncRNA (PU.1 AS lncRNA) promotes adipogenesis by attenuating PU.1 mRNA translation [16,17]. Intramuscular fat-associated long non-coding RNA (lncRNA IMFNCR) promotes intramuscular adipocytes differentiation by sponging miR-27b-3p and miR-128-3p, thus increasing the expression of its target gene peroxisome proliferator activated receptor γ (*PPARγ*) [18]. In recent years, the application of transcriptome sequencing has made it easier to identify a large number of novel lncRNAs in the adipose tissue [19,20]. Sun et al. found 175 adipose-enriched lncRNAs, which were differentially expressed during adipogenesis and were regulated by main transcription factors such as *PPAR*γ and CCAAT/enhancer binding protein α (*C/EBPα*) [21]. De novo sequencing of the human adipose tissue transcriptome found a large number of conserved lncRNAs in brown adipose tissue (BAT) [22].

Although a large number of studies have illustrated the roles of lncRNAs in adipogenesis [23,24], the obesity-related lncRNAs and their roles in *ob/ob* mice remain largely unknown. To analyze the different transcriptomes of lncRNAs in wild-type (WT) and *ob/ob* mice, we used RNA-Sequencing (RNA-Seq) to identify obesity-related lncRNAs in *ob/ob* mice. A total of 2809 lncRNAs were identified in WT and *ob/ob* mice, including 248 novel lncRNAs. Of them, 46 lncRNAs were differentially expressed in WT and *ob/ob* mice. Furthermore, we identified a novel lncRNA, obesity-related lncRNA (lnc-ORA), with a seven-fold change in *ob/ob* mice compared to WT mice, suggesting it may play an important role in adipogenesis. Knockdown of lnc-ORA inhibited preadipocytes proliferation, manifested by a decrease in the mRNA and protein levels of cell cycle markers, including *PCNA, cyclin B, cyclin D1*, and *cyclin E*. Furthermore, knockdown of lnc-ORA inhibited adipocytes differentiation by regulating the PI3K/AKT/mTOR signaling pathway. Collectively, these results not only expand the lncRNA regulation network during adipogenesis but also provide potential molecular therapeutic targets to treat obesity-related diseases.

## 2. Materials and Methods

### 2.1. Ethics Statement

Eight-week-old C57BL/6J male WT mice (n = 5) and *ob/ob* mice (n = 5) were purchased from the Experimental Animal Center of Xi’an Jiao-Tong University. All animal procedures were approved by the Northwest A and F University Animal Care Committee (NWAFU-314020038). 

### 2.2. RNA Extraction, Library Preparation, Sequencing, and qPCR

Adipose tissue samples were isolated from inguinal white adipose tissue (iWAT) of WT and *ob/ob* mice. Total RNA was extracted from iWAT samples by using the TRIzol reagent according to the manufacturer’s instructions (Takara, Kyoto, Japan). RNA degradation and RNA purity were determined according to our previous method [25]. A total amount of 3 μg RNA per pooled sample (n = 5) was used as input material for the preparation of the RNA samples. Firstly, ribosomal RNA (rRNA) was removed by Epicentre Ribo-zero™ rRNA Removal Kit (Epicentre, Madison, WI, USA), and the rRNA-free residue was purified by ethanol precipitation. Subsequently, sequencing libraries were generated using the rRNA-depleted RNA using the NEBNext^®^ Ultra™ Directional RNA Library Prep Kit for Illumina^®^ (NEB, Ipswich, MA, USA), following the manufacturer’s recommendations. The clustering of the index-coded samples was performed on a cBot Cluster Generation System using TruSeq PE Cluster Kit v3-cBot-HS (Illumia, San Diego, California, USA), according to the manufacturer’s instructions. After cluster generation, the libraries were sequenced on an Illumina Hiseq 2500 platform, and 125-bp paired-end reads were generated. Then, 1 μg of total RNA was reversed-transcribed using random primers, according to the manufacturer’s instructions (Takara, Kyoto, Japan), and quantitative PCR (qPCR) was performed using an SYBR-Green kit (Takara, Kyoto, Japan) and the StepOne Plus system for detection. The qPCR primers used are shown in Table 1.

### 2.3. Quality Control

Raw reads of fastq format were firstly processed through in-house perl scripts. In this step, clean reads were obtained by removing reads containing adapter, reads containing ploy-N, and low-quality reads from raw data. At the same time, Q20, Q30, and GC content of the clean data were calculated. All downstream analyses were based on clean, high-quality data.

### 2.4. Mapping to the Reference Genome and Transcriptome Assembly

We used Bowtie v2.0.6 to build an index of the reference genome and TopHat v2.0.9 to align paired-end clean reads to the reference genome (Mus musculus.GRCm38). The mapped reads of each sample were assembled by both Scripture (beta2) and Cufflinks (v2.1.1) in a reference-based approach [26,27]. We then adopted five steps to identify novel lncRNAs from the assembled transcripts: (1) transcripts with a length of <200 bp were removed; (2) transcripts with exon number <2 were removed; (3) transcripts were compared with annotated transcripts using Cuffcompare v2.1.1; (4) transcripts with Fragments Per Kilobase of transcript per Million fragments mapped (FPKM) <0.5 were removed; (5) transcripts that did not pass the protein-coding score test were removed. We used CNCI (coding–non-coding index) (v2) profiles adjoining nucleotide triplets to effectively distinguish protein-coding and non-coding sequences independent of known annotations and the CPC (coding potential calculator) (0.9-r2), which mainly assesses the extent and quality of open reading frames (ORFs) in a transcript and searches the sequences in a known protein sequence database to clarify the coding and non-coding transcripts. We translated each transcript in all three possible frames and used Pfam Scan (v1.3) to identify the occurrence of any of the known protein family domains documented in the Pfam database (using both Pfam A and Pfam B). Any transcript with a Pfam hit was excluded in the following steps. PhyloCSF (phylogenetic codon substitution frequency) (v20121028) was used to examine the evolutionary signatures characteristic of alignments of conserved coding regions, such as the high frequencies of synonymous codon substitutions and of conservative amino acid substitutions as well as the low frequencies of other missense and non-sense substitutions to distinguish protein-coding and non-coding transcripts.

### 2.5. Quantification of Gene Expression Level

FPKM reads of both lncRNAs and coding genes were calculated by Cuffdiff (v2.1.1) in each sample. Gene FPKMs were counted by summing the FPKMs of transcripts in each gene group.

### 2.6. Differential Expression Analysis and KEGG Enrichment Analysis

Cuffdiff was used to determine differential expression in digital transcript or gene expression data by a model based on the negative binomial distribution. The *p*-values were adjusted using the Benjamini and Hochberg method. A corrected *p*-value of 0.05 was set as the threshold for significantly differential expression. We used the KOBAS software to detect the statistical enrichment of differentially expressed lncRNAs and differentially expressed genes (DEGs) in KEGG pathways.

### 2.7. Cell Culture and Transfection

The 3T3-L1 cell line, obtained from American Type Culture Collection (ATCC, Manassas, VA, USA), was cultured in high-glucose Dulbecco’s Modified Eagle Medium (DMEM) with 10% fetal bovine serum (FBS), 100 μg/mL streptomycin, and 100 U/mL penicillin under an atmosphere of 5% CO2 at 37 °C. After two days of culture, when the cells reached confluence, the cells were induced by substituting growth medium (GM) with the differentiation medium (DM), composed of DMEM, 1 μM dexamethasone (Sigma, St. Louis, MO, USA), 10 μg/mL insulin (Sigma), and 0.5 mM 3-isobuty-1-methxlzanthine (IBMX). After two days, the medium was changed with the induction medium containing 10% FBS and 10 μg/mL insulin. For proliferating preadipocytes transfection, preadipocytes were seeded in 6-well or 12-well plates, and lncRNA siRNA or the negative control (NC) (Ribobio, Guangzhou, China) at a concentration of 50 nM was transfected into cells at 40% density, using X-tremeGENE siRNA Transfection Reagent (Roche, Basel, Switzerland) and Opti-MEM (Gibco, Grand Island, NY, USA) culture medium, according to the manufacturer’s protocol. The sequence of NC was 5′-UUCUCCGAACGUGUCACGUTT-3′, 5′-ACGUGACACGUUCGGAGAATT-3′; the sequence used for siRNA was 5′-GGAGAUCUAACUAGUUACATT-3′, 5′-UGUAACUAGUUAGAUCUCCTT-3′. The cells were harvested 24 h or 48 h after transfection. For adipocytes differentiation studies, the cells were transfected when their density reached 70–80%; the transfection protocol was the same as for proliferating preadipocytes transfection. 

### 2.8. Western Blot Analysis and BODIPY Staining 

Protein extraction and Western blot analysis of tissues and cells were performed according to our previous methods [14]. Antibodies against PCNA, cyclin B, cyclin D1, cyclin E, p27, leptin, and PGC-1α were from Santa Cruz Biotechnology. Antibodies against p-PI3K, PI3K, p-AKT, AKT, p-mTOR, mTOR, p-ERK, ERK, p-p38, and p38 were from Cell Signaling Technology. Antibodies against FASN (BOSTER, wuhan, China), FABP4 (BOSTER, China), PPARγ (abcam, Cambridge, MA, USA), and β-tubulin (Boster, China) were used. BODIPY staining was performed according to our published method; the intensity of fluorescence was analyzed by ImageJ [14].

### 2.9. RNA-Fluorescent In Situ Hybridization

The subcellular localization of lnc-ORA was detected by a fluorescent in situ hybridization (FISH) kit (Ribobio, China) according to the manufacturer’s instruction. Briefly, the preadipocytes were washed with PBS and fixed in 4% paraformaldehyde for 10 min. Then, the cells were permeabilized with a 0.5% Triton X-100 solution at 4 °C for 5 min, washed with PBS three times for 5 min, and pre-hybridized at 37 °C for 30 min. Then, an oligodeoxynucleotide probe of anti-lnc-ORA, anti-18s, or anti-U6 was used in the hybridization solution at 37 °C overnight in the dark. The next day, the cells were stained with DAPI and imaged using a confocal laser-scanning microscope.

### 2.10. Cytoplasmic and Nuclear RNA Extraction

For the detection of the lnc-ORA location in cytoplasmic and nuclear RNA, cultured 3T3-L1 preadipocytes were washed with PBS, suspended in lysis buffer, and then incubated on ice for 5 min. After centrifuging at 8000 rpm for 5 min, the supernatant was transferred to a new microcentrifuge tube and subjected to cytoplasmic RNA extraction, while the pellet was resuspended in lysis buffer and subjected to nuclear RNA extraction. For RNA extraction, the fractions were first incubated with Proteinase K (10 mg/mL) at 37 °C for 20 min and then mixed with Trizol (Carsbad, CA, USA). RNA was separated by chloroform and precipitated by ethanol with 3 M sodium acetate [28].

### 2.11. Rapid Amplification of 5′ and 3′ cDNA Ends (RACE)

RACE was performed using the SMARTer RACE Kit with SMARTer II A Oligonucleotide and SMARTScribe™ Reverse Transcriptase (Takara) according to the instruction of the manufacturer. Ex Taq and LA Taq with GC Buffer (Takara) were used for PCR amplification following the manufacturer’s protocol. PCR products were gel-purified with QIAquick (QIAGEN, Duesseldorf, Germany), cloned into the pGEM-T easy vector, and sequenced. The primer used for 5′*RACE* was 5′-GATTACGCCAAGCTTCCTTCAGCCCATCTTGTGTCCCGT-3′. The primer for 3′*RACE* was 5′-GATTACGCCAAGCTTGGGACACAAGATGGGCTGAAGGGG-3′. 

### 2.12. Flow Cytometry, Edu, and CCK-8 Assays

Flow cytometry, Edu staining, and cell count kit 8 (CCK-8) assays were performed according to our previously described method [29]. Preadipocytes were transfected with NC and siRNA when the cell densities were 40%. Then, 24 h or 48 h after transfection, the preadipocyte proliferation was assessed.

### 2.13. Statistical Analysis

Statistical analysis was performed using Graph Pad Prism 6 (Graph pad software, La Jolla, CA, USA). Comparisons among the individual means were made by a Student’s t test. Data are presented as the mean ± SEM. A *p* value <0.05 was considered significant.

## 3. Results

### 3.1. Identification and Characterization of lncRNAs in WT and ob/ob Mice

**Figure 1:**
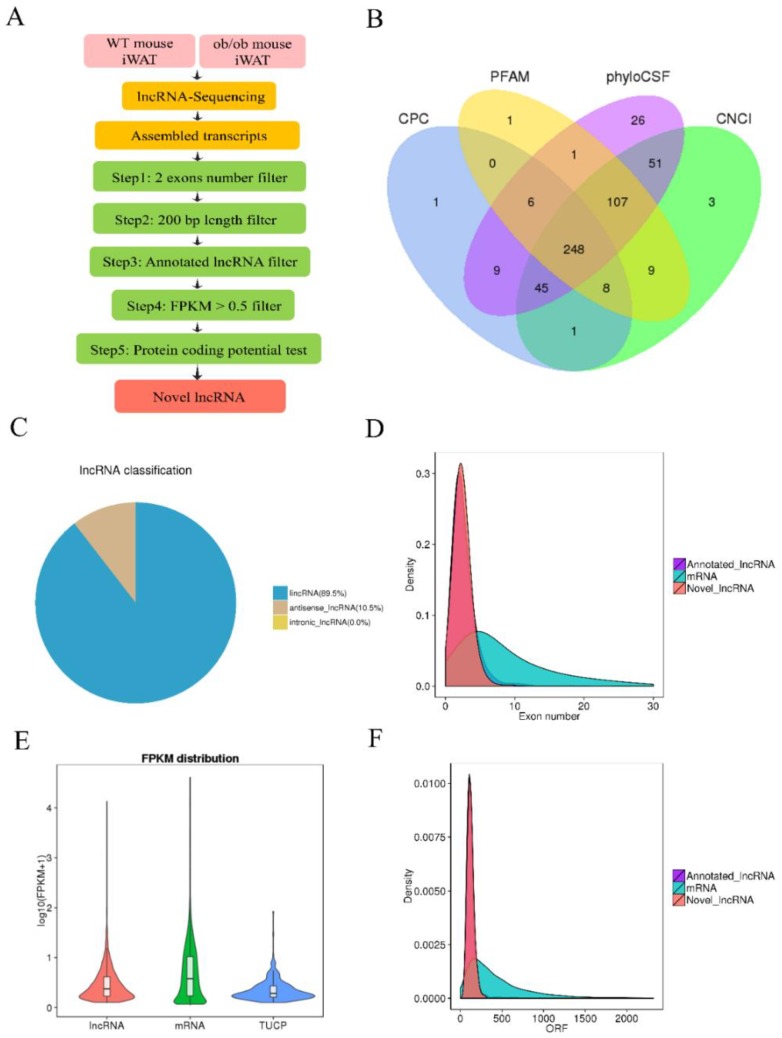
Identification and characterization of long noncoding RNAs (lncRNAs) in wild-type (WT) and *ob/ob* mice. (**A**) Flow chart of novel lncRNAs identification in WT and *ob/ob* mice. (**B**) Venn diagram of coding potential analysis by the coding potential calculator (CPC), the Pfam Scan, phylogenetic codon substitution frequency (phyloCSF), and the coding–non-coding index (CNCI). (**C**) Pie charts of the lncRNA classification, including lincRNA, antisense lncRNA, and intronic lncRNA. (**D**) Density of transcript exon number. (**E**) Fragments Per Kilobase of transcript per Million fragments mapped (FPKM) reads distribution of transcripts. (**F**) Open reading frame (ORF) length of transcripts. iWAT: inguinal white adipose tissue.

To illustrate the differential lncRNA profiles in iWAT between WT and *ob/ob* mice and to identify obesity-related lncRNAs, we performed RNA-seq of iWAT samples, which were isolated from eight-week-old WT and *ob/ob* mice kept with identical feeding conditions. The identification flow chart of novel lncRNAs is presented in Figure 1A. In total, 248 novel lncRNAs were identified in WT and *ob/ob* mice after transcript-coding potential analysis of the four prediction methods: the coding potential calculator (CPC), Pfam Scan database, phylogenetic codon substitution frequency (phyloCSF), and the coding–non-coding index (CNCI) (Figure 1B). The lncRNA classification showed that 89.5% of novel lncRNAs transcripts were long intergenic noncoding RNAs (lincRNA), and 10.5% of novel lncRNAs transcripts were antisense lncRNA (AS lncRNAs); there were no intronic lncRNAs (Figure 1C). Moreover, the number of exons of annotated and novel lncRNAs was less than that of mRNAs (Figure 1D). The expression levels of lncRNAs were slightly lower than those of mRNAs (Figure 1E). In addition, the ORF lengths of lncRNAs were shorter than those of mRNAs (Figure 1F).

### 3.2. Differentially Expressed lncRNAs and Genes in WT and ob/ob Mice

**Figure 2:**
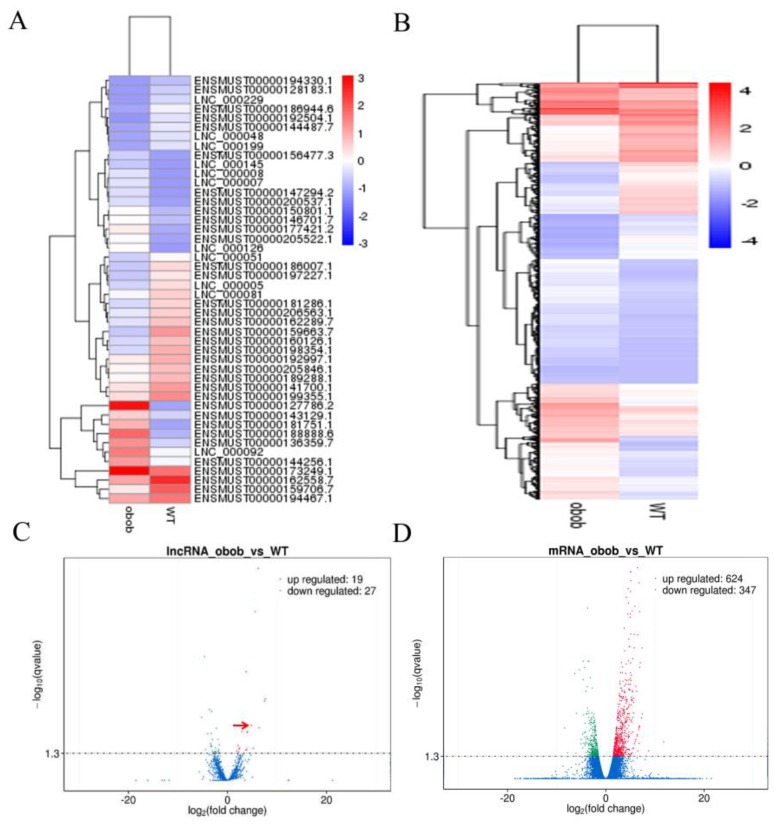
Differentially expressed lncRNAs and differentially expressed genes (DEGs) in WT and *ob/ob* mice. (**A**) Heat map of the differentially expressed lncRNAs in WT and *ob/ob* mice (fold change ≥2 and *q* value ≤0.05). (**B**) The heat-map of the differentially expressed mRNAs in WT and *ob/ob* mice (fold change ≥ 2; *q*-value ≤ 0.05). (**C**) Volcano plot of differentially expressed lncRNAs. Among these lncRNAs, 19 lncRNAs were upregulated and 27 lncRNAs were downregulated in *ob/ob* mice. lnc-ORA was denoted by red arrow. (**D**) Volcano Plot of differentially expressed mRNAs. Among these mRNAs, 624 mRNAs were upregulated and 347 mRNAs were downregulated in *ob/ob* mice.

To screen the DEGs and differentially expressed lncRNAs in WT and *ob/ob* mice, Cuffdiff v2.1.1 was used to analyze differential expression levels of lncRNAs and mRNAs. The results showed that there were 46 differentially expressed lncRNAs and 971 DEGs in WT and *ob/ob* mice, respectively (Figure 2A,B). Next, a volcano plot analysis found that there were 19 upregulated and 27 downregulated lncRNAs, and 624 upregulated and 347 downregulated DEGs in the iWAT of *ob/ob* mice (Figure 2C,D).

### 3.3. Pathway Analysis of DEGs

**Figure 3:**
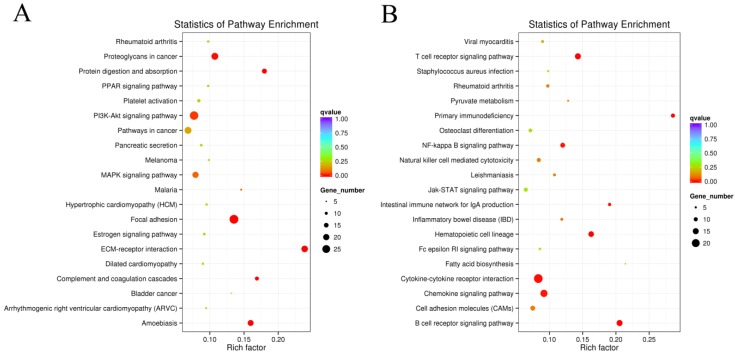
KEGG analysis of the DEGs. (**A**) Top 20 upregulated signaling pathways in KEGG enrichment analysis in *ob/ob* mice. (**B**) Top 20 downregulated signaling pathways in KEGG enrichment analysis in *ob/ob* mice.

In order to further confirm the potential function of obesity-related lncRNAs in adipogenesis, KEGG analyses of the DEGs were performed. The results showed that the DEGs were enriched in 122 signaling pathways. The top 20 upregulated and top 20 downregulated signaling pathways are shown, respectively, in Figure 3A,B. The results showed that many pathways related to fat metabolism and adipogenesis were significantly enriched. Of them, the PI3K/AKT signaling pathway had the highest enrichment, and the cytokine–cytokine receptor interaction showed the greatest downregulation, suggesting that the two signaling pathways may be associated with obesity-induced lncRNAs in adipogenesis.

### 3.4. Validation of the Differentially Expressed lncRNAs by qPCR

**Figure 4:**
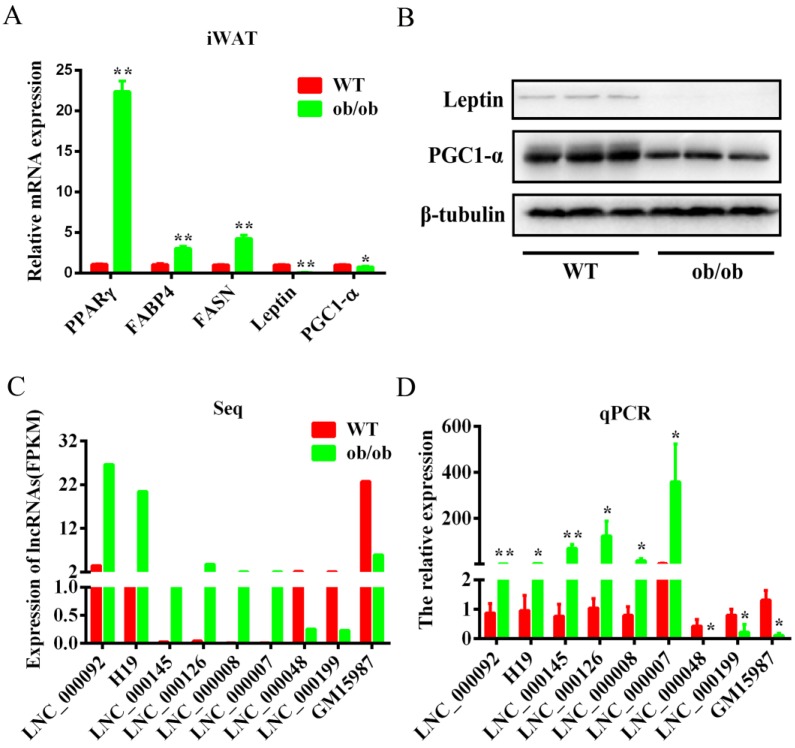
Validation of the differentially expressed lncRNAs by qPCR. (**A**) mRNA expression levels of adipogenic marker genes in WT and *ob/ob* mice. (**B**) Protein expression levels of leptin and PGC1-a in the iWAT of WT and *ob/ob* mice. (**C**) Sequencing FPKM values of nine lncRNAs. (**D**) The lncRNAs were validated by qPCR assays. Data are expressed as the mean ± SEM (n = 5). * *p*< 0.05, ** *p* < 0.01.

The adipogenic ability of obese mice was obviously higher than that of WT mice. First, we detected the expression levels of several adipogenic marker genes in the iWAT of WT and *ob/ob* mice, such as *PPAR*γ, fatty acid binding protein 4 (*FABP4*), fatty acid synthase (*FASN*), *leptin*, and peroxisome proliferator-activated receptor gamma coactivator 1-α (*PGC-1α*) (Figure 4A). The results showed that the expression levels of *PPAR*γ, *FABP4*, and *FASN* in *ob/ob* mice were higher than in WT mice. However, the expression levels of the thermogenesis gene *PGC1-α* was lower than in WT mice (Figure 4B), indicating that our sequencing samples were suitable for an obesity model. To confirm the accuracy of the sequencing data, nine randomly selected lncRNAs were validated by qPCR. The results showed that the expression patterns of lncRNAs were similar to those of the sequencing data (Figure 4C,D). Specifically, we found that LNC_0000092, the newly named lnc-ORA (obesity-related lncRNA), was abundant in the iWAT of *ob/ob* mice, with a seven-fold expression change compared to its expression in WT mice, suggesting it may be an important lncRNA during adipogenesis.

### 3.5. Identification, Subcellular Locations, and Expression Pattern Analysis of lnc-ORA

**Figure 5:**
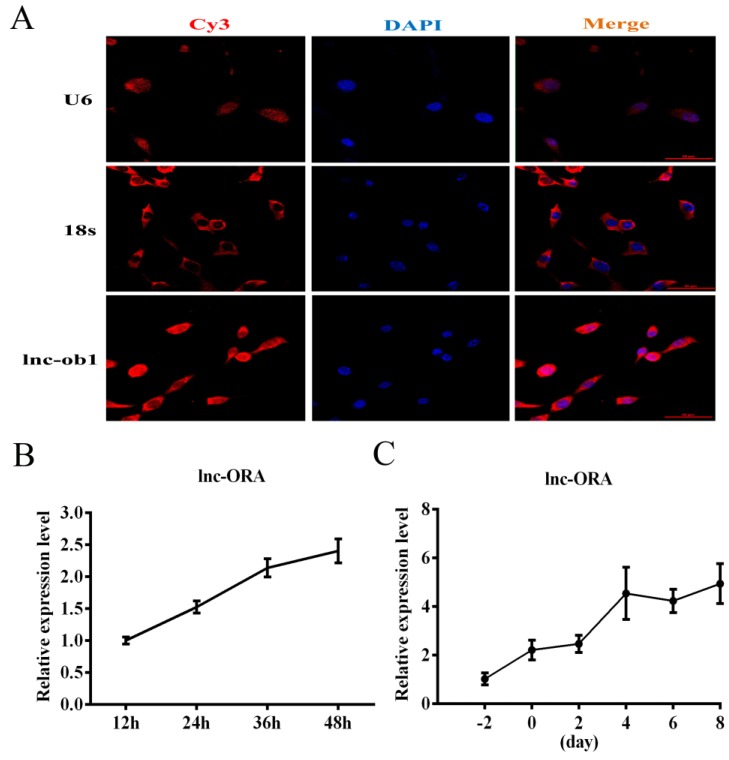
Subcellular locations and expression pattern analysis of lnc-ORA. (**A**) Subcellular localization of lnc-ORA by fluorescent in situ hybridization (FISH) in preadipocytes. Special FISH probes against lnc-ORA, U6, and 18s were modified by Cy3 (red); 18S is a cytoplasmic marker, and U6 is a nuclear marker. The nucleus was stained by DAPI (blue). (**B**) Expression pattern of lnc-ORA during preadipocytes proliferation. (**C**) Expression levels of lnc-ORA after inducing adipocytes differentiation. Data are expressed as the mean ± SEM (n = 3).

lnc-ORA was transcribed from locus 89,767,653 to locus 89,768,886 on chromosome 15 and consisted of two exons and one intron. The alignment track showed that lnc-ORA is an unannotated lncRNA in the mouse genome (Figure S1A). The rapid amplification of cDNA ends (RACE) and PCR experiments indicated that the full length of lnc-ORA was 836 bp (Figure S1B–D). To confirm the subcellular locations of lnc-ORA in preadipocyte, FISH, and cytoplasmic and nuclear RNA separated extraction experiments were performed. The results showed that lnc-ORA was expressed in both cytoplasmic and nuclear locations (Figure 5A and Figure S2B). Then, we used the Coding Potential Assessment Tool (CPAT) to evaluate the protein-coding potential of lnc-ORA. The results showed that lnc-ORA had no protein-coding potential (Figure S2A). The cell-specific localization of lnc-ORA in adipose tissue (AT) was determined by the isolation of preadipocytes, macrophages, and adipocytes from the AT of obese mice. The results showed that lnc-ORA was mainly expressed in adipocytes and preadipocytes and almost not expressed in macrophages (Figure S2C). lnc-ORA was expressed in various tissues, including heart, liver, spleen, kidney, BAT, and muscle and was expressed at the highest levels in WAT compared to other tissues (Figure S2D). 3T3-L1 cells were differentiated and generated lipid droplets after 8 d induction (Figure S3A), which is consistent with the expression of adipogenic marker genes (Figure S3B–D). Moreover, the expression levels of lnc-ORA gradually increased during proliferation and differentiation of 3T3-L1 cells (Figure 5B,C), indicating that this lncRNA could regulate preadipocytes’ proliferation and differentiation during lipid formation.

### 3.6. Knockdown of lnc-ORA Inhibits the Proliferation of Preadipocytes

**Figure 6:**
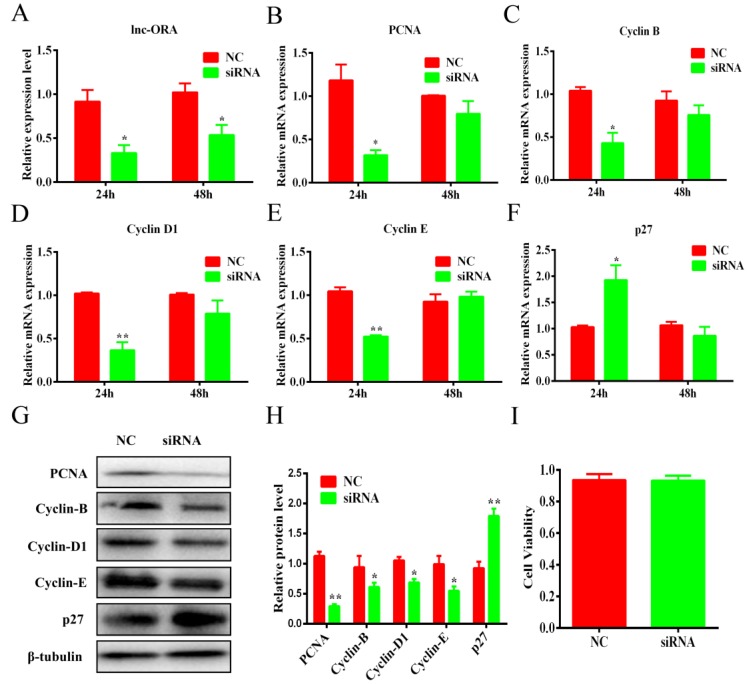
Knockdown of lnc-ORA downregulates the expression levels of cell cycle genes. (**A**) Knockdown efficiency of lnc-ORA after preadipocytes transfection for 24 h and 48 h. (**B**–**F**) The mRNA expression of cell cycle marker genes *PCNA, cyclin B, cyclin D1, cyclin E*, and *p27* was detected by qPCR. (**G**) Protein expression of cell cycle marker genes *PCNA*, *cyclin B, cyclin D1*, *cyclin E*, and *p27* was detected by Western blot. (**H**) Protein quantitative analysis of PCNA, cyclin B, cyclin D1, cyclin E, and p27. (**I**) CCK-8 analysis of cell viability of transfected preadipocytes. Data are expressed as the mean ± SEM (n = 3). * *p* < 0.05, ** *p* < 0.01, vs. NC.

**Figure 7:**
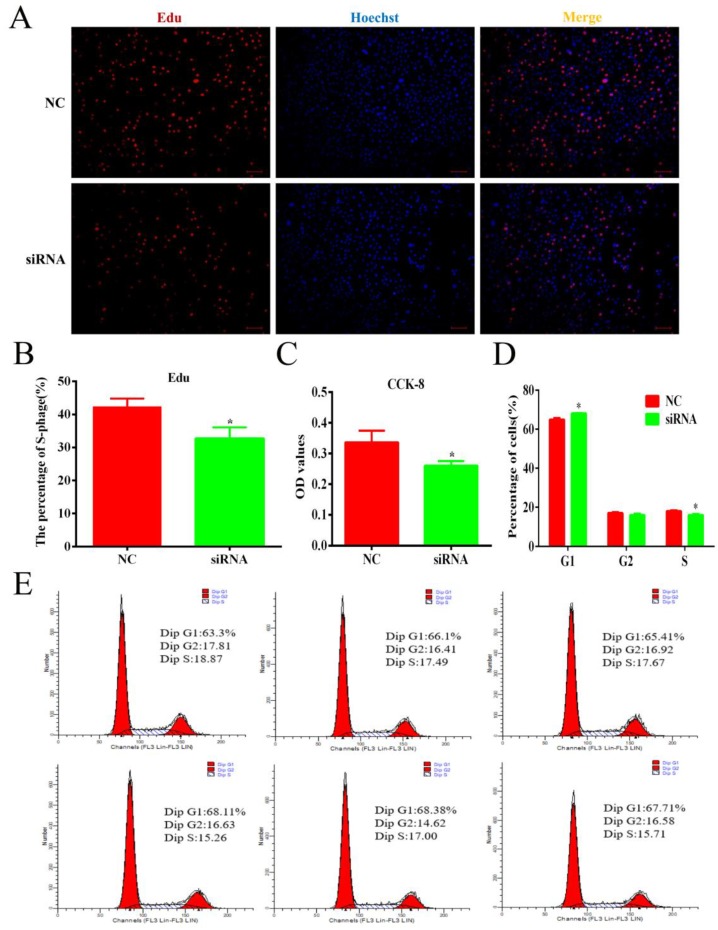
Knockdown of lnc-ORA inhibits cell proliferation and decreases the number of total cells as well as of S-phase cells. (**A**) Edu staining analysis of preadipocytes. Preadipocytes in the S-phase were stained with Edu in red, while the cell nuclei were dyed with Hoechst in blue. (**B**) Percentage of Edu-positive cells vs. total cells. (**C**) CCK-8 analysis after treatment with siRNA during preadipocyte proliferation. (**D**) Statistical analysis of flow cytometry data. (**E**) Flow cytometry analysis of preadipocytes. Data are expressed as the mean ± SEM (n = 3). * *p* < 0.05, vs. NC.

To confirm the role of lnc-ORA in preadipocyte proliferation, 3T3-L1 cells were transfected with siRNA or an NC. The results showed that the expression levels of lnc-ORA obviously decreased after transfection for 24 h and 48 h (Figure 6A). Cell viability was not significantly affected by siRNA transfection (Figure 6I). The mRNA levels of cell cycle marker genes, including proliferating cell nuclear antigen (*PCNA*) (Figure 6B), cyclin-dependent kinases B (*cyclin B*) (Figure 6C), *cyclin D1* (Figure 6D), and *cyclin E* (Figure 6E), were significantly decreased after transfection for 24 h but significantly increased the mRNA levels of cyclin-dependent kinase inhibitor 1B (*p27*) (Figure 6F) and were consistent with the protein levels (Figure 6G,H). Furthermore, the Edu staining assay indicated that knockdown of lnc-ORA decreased the number of Edu-labeled cells (Figure 7A,B). CCK-8 showed that the total cell number after lnc-ORA knockdown was less than the number of NC-treated cells (Figure 7C). In addition, the knockdown of lnc-ORA decreased the number of cells in S-phase and increased the number of cells in G1-phase, as determined by flow cytometer analysis (Figure 7D,E), indicating that the knockdown of lnc-ORA inhibited preadipocytes proliferation, possibly through the suppression of the DNA replication process.

### 3.7. Knockdown of lnc-ORA Inhibits Adipocytes Differentiation through the PI3K/AKT/mTOR Signaling Pathway

**Figure 8:**
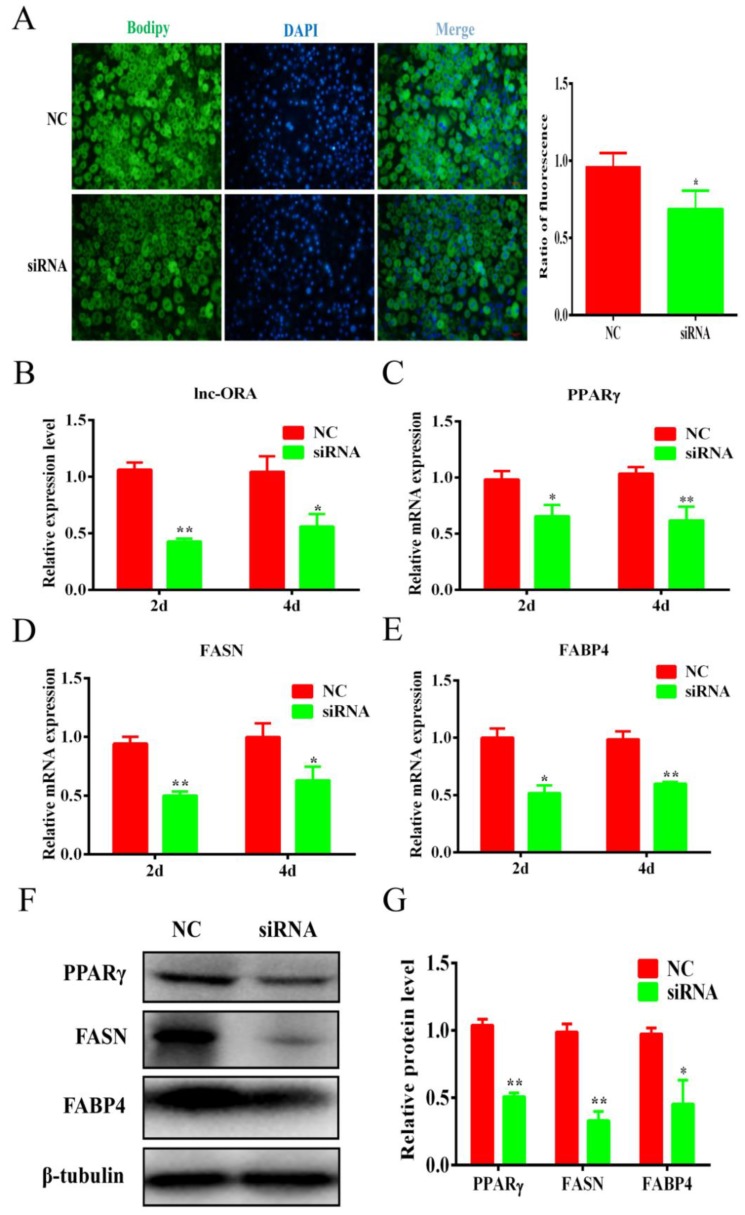
Knockdown of lnc-ORA inhibits adipocytes differentiation. (**A**) BODIPY staining of adipocytes after induction for 6 d. (**B**) Knockdown efficiency of lnc-ORA after adipocyte transfection for 2 d and 4 d. (**C**–**E**) mRNA levels of adipocytes markers, including *PPAR*γ*, FASN*, and *FABP4,* after adipocyte transfection for 2 d and 4 d. (**F**) Protein levels of adipocyte-specific genes, including PPARγ, FASN, and FABP4, analyzed by Western blot. (**G**) Protein quantification analysis by Western blot. Data are expressed as the mean ± SEM (n = 3). * *p* < 0.05, ** *p* < 0.01, vs. NC.

**Figure 9:**
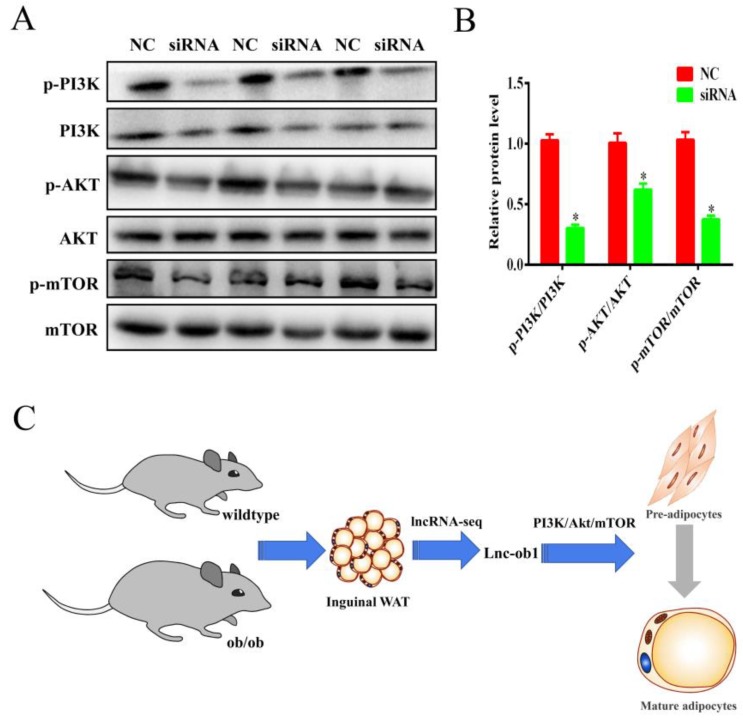
lnc-ORA regulates adipogenesis through the PI3K/AKT/mTOR signaling pathway. (**A**) The activity of the signaling pathway was analyzed by Western blot after siRNA treatment. The protein levels of p-PI3K, PI3K, p-AKT, AKT, p-mTOR, and mTOR were detected by Western blot. (**B**) Protein quantification analysis by Western blot. (**C**) Flow diagram showing how lnc-ORA regulates adipogenesis. Data are expressed as the mean ± SEM (n = 3). * *p* < 0.05, vs. NC.

To explore the function of lnc-ORA regulation on adipogenic differentiation, 3T3-L1 cells were transfected with siRNA or NC at a confluence of 70–80%. The expression of lnc-ORA was decreased to 30–40% by siRNA-mediated lnc-ORA knockdown (Figure 8B). BODIPY staining indicated that the knockdown of lnc-ORA inhibited lipid accumulation in 3T3-L1 cells (Figure 8A). The knockdown of lnc-ORA significantly reduced the mRNA expression levels of the adipogenesis markers *PPAR*γ*, FASN*, and *FABP4* at Days 2 and 4 (Figure 8C–E), which was consistent with the protein levels (Figure 8F,G). Collectively, these data demonstrated that the knockdown of lnc-ORA markedly inhibited adipocytes differentiation. In addition, we further examined the PI3K/AKT/mTOR, ERK, and p38 signaling pathways during adipogenesis. The results showed that the knockdown of lnc-ORA reduced the ratios of p-PI3K/PI3K, p-AKT/AKT, and p-mTOR/mTOR during adipocyte differentiation, whereas the ERK and p38 signaling pathways were not affected (Figure 9A,B and Figure S4), indicating that lnc-ORA regulates adipocytes differentiation through the PI3K/AKT/mTOR signaling pathway (Figure 9C).

## 4. Discussion

In this study, a total of 2809 lncRNA transcripts were identified in the iWAT from WT and *ob/ob* mice, of which 248 were novel lncRNAs. Further, 46 differentially expressed lncRNAs in WT and *ob/ob* mice were identified. Specifically, a novel lncRNA, named lnc-ORA, enhanced preadipocytes proliferation and adipogenesis through the PI3K/AKT/mTOR signaling pathway. Overall, our findings provide direct evidence that lnc-ORA is a novel positive regulator of adipogenesis.

Many different processes contribute to the commitment of a mesenchymal stem cell to the adipocyte lineage, including the regulation of a complex network of transcription factors, cofactors, and numerous signaling pathways [30,31,32,33,34,35]. Alvarez-Dominguez et al. used RNA-seq to reconstruct de novo transcriptomes of mouse BAT, iWAT, and eWAT and identified lnc-BATE1 as a regulator of BAT development [36]. Lo et al. performed an analysis of lncRNA transcriptome in adipocytes isolated from BAT and WAT in high-fat-diet-induced obese mice. The results identified a series of obesity-related lncRNAs, among which the most crucial was lnc-leptin, transcribed from the promoter of leptin. lnc-leptin promoted adipogenesis through direct interaction between its DNA loci and leptin [8]. Our previous study investigated the difference of lncRNAs in fat-type bamei pigs and lean-type large white pigs [25]. Although many studies have investigated lncRNAs related to obesity [37,38,39], the different expression of lncRNAs in WAT between *ob/ob* and WT mice is unclear. Here, we identified 248 novel lncRNAs in WT and *ob/ob* mice. The characterization of the novel lncRNAs was consistent with basic features of lncRNA, as determined by analyzing exon number, FPKM distribution, and ORF length. Specifically, we focused on an obesity-related lncRNA, lnc-ORA, which showed higher expression in the adipose tissue and more than seven-fold upregulation in *ob/ob* mice compared with WT mice. Given the higher expression levels in the adipose tissue, we used the knockdown method to eliminate its function in 3T3-L1 cells. Interestingly, the results showed that the knockdown of lnc-ORA inhibited both preadipocyte proliferation and processes to alleviate adipogenesis, as indicated by a decrease in the number of cells in S-phase and the expression of cell cycle marker genes *PCNA, cyclin B, cyclin D1*, and *cyclin E*, as well as by a decrease in the expression of adipogenesis markers *PPAR*γ, *FASN*, and *FABP4*. The mitotic clonal expansion (MCE) is a prerequisite for adipocyte differentiation that occurs within 48 h of adipogenic stimulation [40]. After induction of differentiation, postconfluent and growth-arrested 3T3-L1 preadipocytes synchronously reenter the cell cycle, undergo several rounds of mitotic clonal expansion, and then express genes that produce the adipocyte phenotype [41]. Here, we found that knockdown of lnc-ORA inhibited preadipocyte proliferation by analysis of flow cytometry, Edu, CCK-8, and the levels of proliferating key genes, implying lnc-ORA may be involved in the mitotic clonal expansion phase and hence impair terminal adipocyte differentiation. These data indicate that lnc-ORA is a positive regulator of adipogenesis and provide an explanation for its higher levels in *ob/ob* mice than in WT mice.

lncRNAs not only regulate the transcription of target genes in the nucleus but also participate in the post-transcriptional regulation of genes in the cytoplasm, so the subcellular localization of lncRNAs is important for elucidating their function [42,43]. We found that lnc-ORA was located in both the nucleus and the cytoplasm of preadipocytes, implying that lnc-ORA could participate in target gene regulation at both the transcription and the post-transcription levels.

The leptin-deficient *ob/ob* mouse is a key animal model for the investigation of obesity and type 2 diabetes. Leptin receptors are members of the cytokine family of receptors. The long-form receptor has been shown to activate the STAT, MAPK, and PI3K signaling pathways [44,45,46]. In addition, it has been demonstrated that PI3K participates in obesity-related biological processes [47]. Leptin-stimulated secretion of insulin requires calcium and the activation of the PI3K/AKT signaling pathway [48,49,50]. Previous studies have indicated that leptin acts on POMC and NPY/AgRP neurons to suppress food intake and promote energy expenditure [51]. One mechanism is leptin-induced PI3K signaling regulation of BAT thermogenesis and WAT browning [52]. PI3Ks are members of a unique and conserved family of enzymes responsible for the phosphorylation of proteins. The insulin-mediated PI3K/AKT signaling pathway plays an important role within adipocytes of obese patients and leads to an excess of lipids that has to be properly stored in the fat tissue [53]. In this study, KEGG analyses of DEGs were performed. We predicted that the DEGs were enriched in 122 signaling pathways. The top 20 upregulated and downregulated KEGG pathways were involved in fat metabolism and adipogenesis processes. Among them, the PI3K/AKT signaling pathway had the highest enrichment, and the cytokine–cytokine receptor interaction showed the greatest downregulation, suggesting that the two signaling pathways may play important roles during adipogenesis. Therefore, we further examined the PI3K/AKT/mTOR, ERK, and p38 signaling pathways after knockdown of lnc-ORA in 3T3-L1 cells. We found that knockdown of lnc-ORA only decreased the ratios of p-PI3K/PI3K, p-AKT/AKT, and p-mTOR/mTOR during adipocytes differentiation, indicating that lnc-ORA is required for adipocytes differentiation through the PI3K/AKT/mTOR signaling pathway. The findings further confirmed our prediction on the basement of KEGG analysis of the DEGs. However, we here must point out that other undetected adipogenic-related signaling pathways may be involved in adipogenic regulation of lnc-ORA.

## 5. Conclusions

In conclusion, we found 46 differentially expressed lncRNAs in the iWAT of *ob/ob* and WT mice and identified a novel lnc-ORA that regulated adipogenesis through the PI3K/AKT/mTOR signaling pathway. These findings contribute to a better understanding of adipogenesis by lncRNAs and provide novel potential therapeutic targets for obesity-related metabolic diseases.

## Figures and Tables

**Table 1:** Primers used for real-time quantitative PCR (qPCR).

Name	Forward (5′→3′)	Reverse (5′→3′)
lnc-ORA	GCCTTGCTTGTGCAGGTCTA	GTCTAGGAAGACTGGGTGCTG
*PPAR*γ	CCAAGAATACCAAAGTGCGATCA	CCCACAGACTCGGCACTCAAT
*FASN*	AATCGGCAAATTCGACCTTTC	ACCTGGATGACCACTTTGCCTAT
*FABP4*	AAGAAGTGGGAGTGGGCTTTG	CTCTTCACCTTCCTGTCGTCTG
*Leptin*	GAGACCCCTGTGTCGGTTC	CTGCGTGTGTGAAATGTCATTG
*Adiponectin*	GGCAGGAAAGGAGAACCTGG	AGCCTTGTCCTTCTTGAAGAG
*cyclin B*	AATCCCTTCTTGTGGTTA	CTTAGATGTGGCATACTTG
*cyclin E*	CAGAGCAGCGAGCAGGAGC	GCAGCTGCTTCCACACCACT
*cyclin D1*	TAGGCCCTCAGCCTCACTC	CCACCCCTGGGATAAAGCAC
*GAPDH*	TGCTGAGTATGTCGTGGAGTCT	ATGCATTGCTGACAATCTTGAG

## Supplementary Materials

The supplementary materials are available online https://www.mdpi.com/2073-4409/8/5/477/s1.
