# Supplementary material for: A Novel lnc-RNA, Named lnc-ORA, Is Identified by RNA-Seq Analysis, and Its Knockdown Inhibits Adipogenesis by Regulating the PI3K/AKT/mTOR Signaling Pathway
Source: Cells. 2019 May 18;8(5):477. doi: 10.3390/cells8050477 (PMC6562744; doi:10.3390/cells8050477)
Supplement: Supplementary file 1 [file cells-08-00477-s001.pdf]

cells

MDPI

A Novel lnc-RNA, Named lnc-ORA, Is Identified by RNA-Seq Analysis, and Its Knockdown Inhibits Adipogenesis by Regulating the PI3K/AKT/mTOR Signaling Pathway

Rui Cai, Guorong Tang, Que Zhang, Wenlong Yong, Wanrong Zhang, Junying Xiao, Changsheng Wei, Chun He, Gongshe Yang, and Weijun Pang *

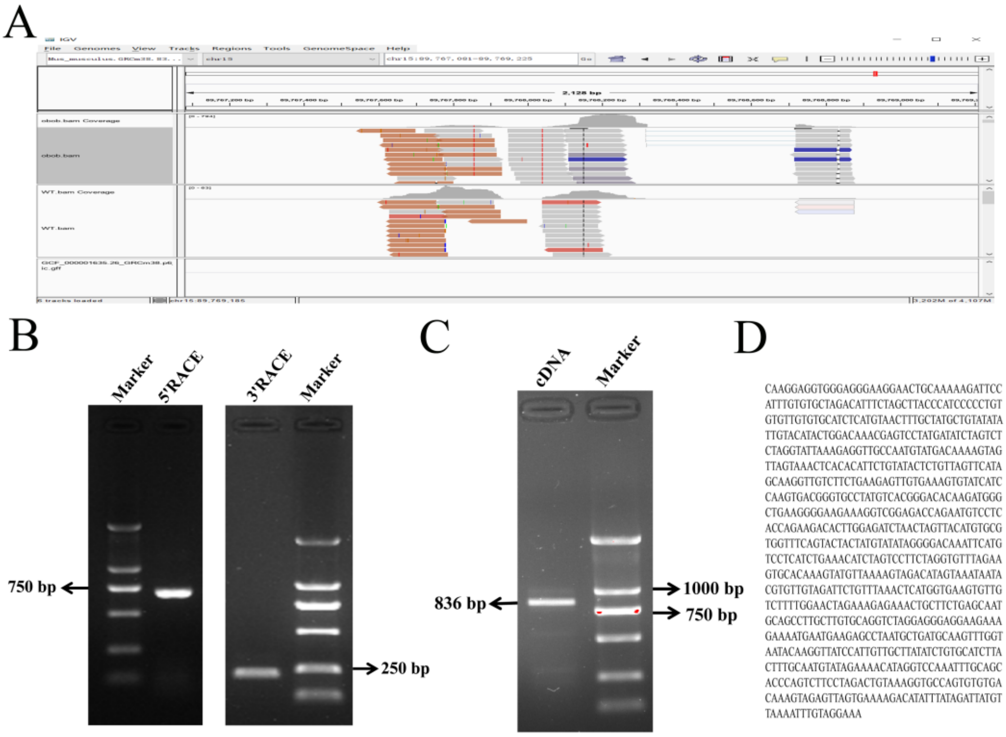

Figure S1. Alignment track, and transcriptional identification of lnc-ORA in mouse. (A) The alignment track of lnc-ORA. (B) Amplification product by Rapid Amplification of cDNA Ends (RACE). (C) Detection of lnc-ORA cDNA using PCR in mouse adipocytes. (D) The sequence information of lnc-ORA.

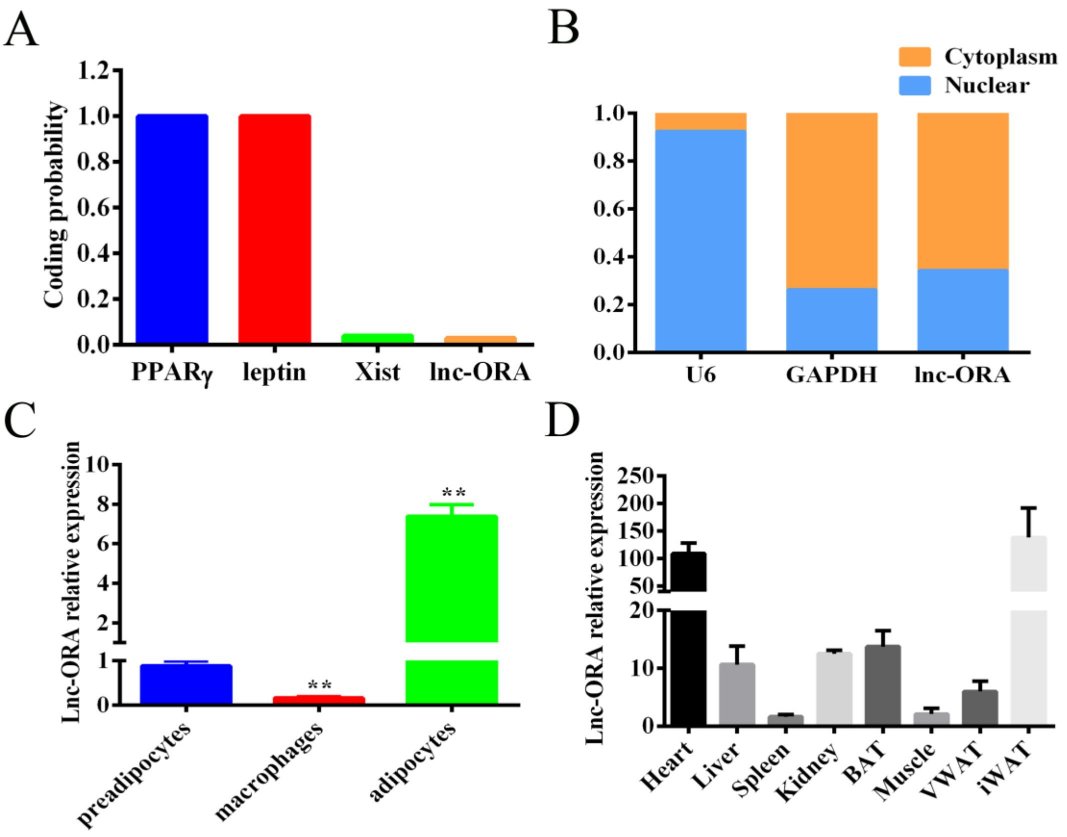

Figure S2. The biological characteristics of lnc-ORA. (A) Coding potential of lnc-ORA was predicted by CAPT analysis. (B) The lnc-ORA expression in nuclear and cytoplasmic fractions extracted from preadipocytes. (C) The expression profile of lnc-ORA in preadipocytes, macrophages and adipocytes. (D) The expression level analysis of lnc-ORA in different tissues of mice.

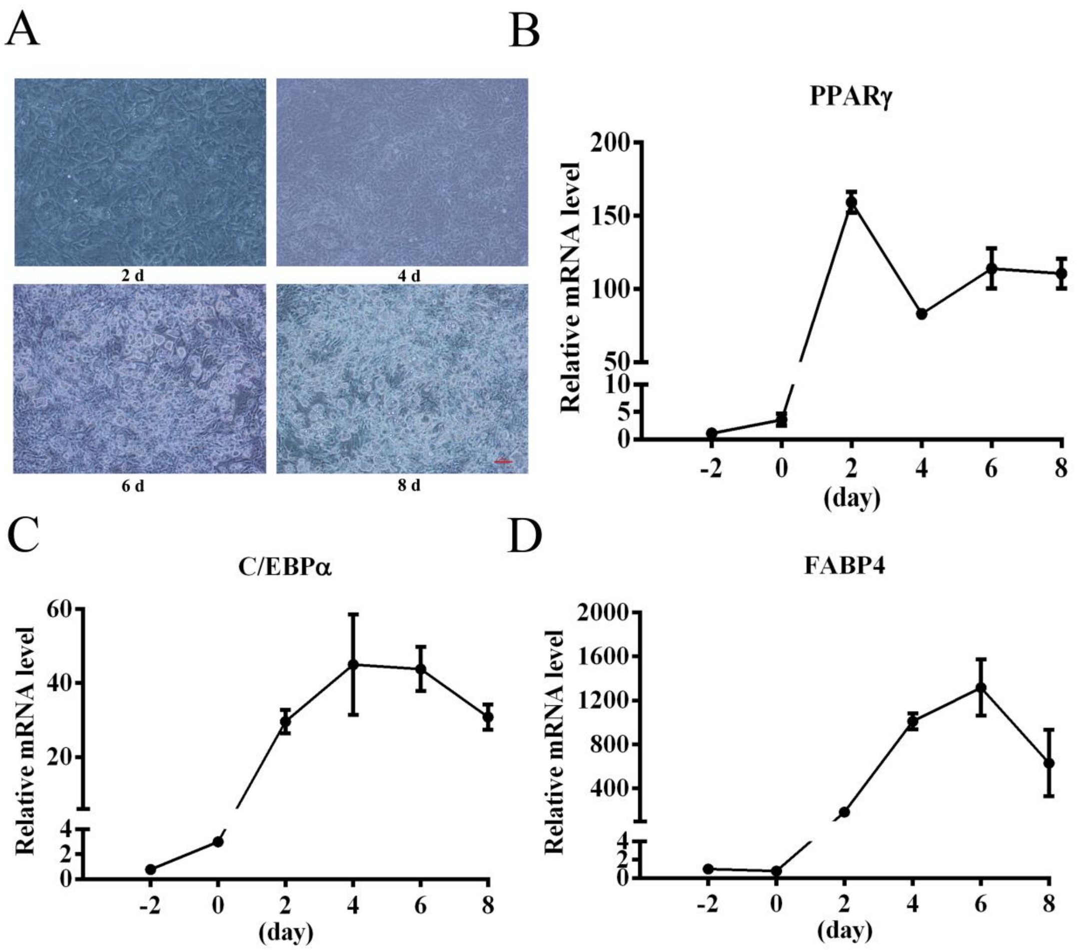

Figure S3. The expression pattern of lnc-ORA during 3T3-L1 cell adipogenesis. (A) The 2 d, 4 d, 6 d and 8 d morphology of 3T3-L1 cells. (B-D) The profiling of adipogenic marker genes PPARγ, C/EBPα, and FABP4 during 3T3-L1 cell differentiation.

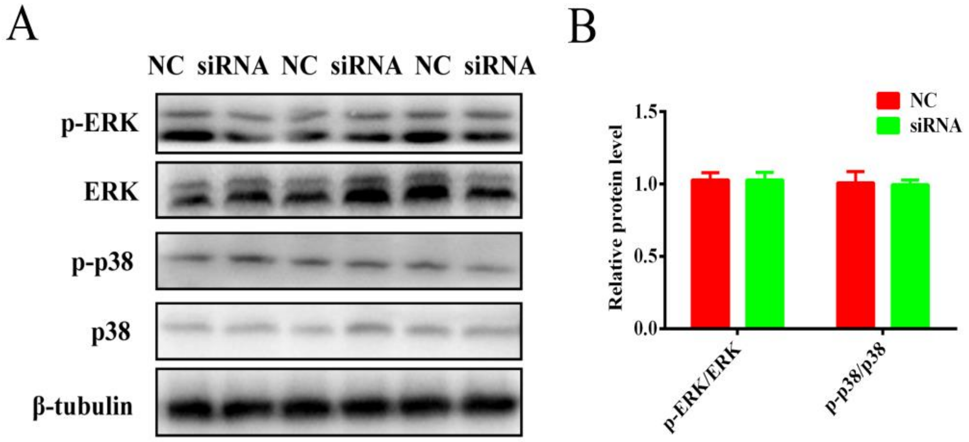

Figure S4. ERK and p38 signaling pathways were not implicated in the regulatory adipogenesis of lnc-ORA knockdown. (A) The protein levels of p-ERK, ERK, p-p38, and p38 were detected by western blot. (B) The protein quantification analysis.
